# ELM-AdaBoost-Based Recognition of Risky Driving Behavior

**DOI:** 10.3390/s26144331

**Published:** 2026-07-08

**Authors:** Dudu Guo, Entong Liu, Guoliang Chen, Miao Sun, Meng Li, Zhenxun Wei

**Affiliations:** 1 Xinjiang Key Laboratory of Green Construction and Smart Traffic Control of Transportation Infrastructure, Xinjiang University, Urumqi 830017, China; 2 Xinjiang Key Laboratory of Highway Engineering Technology in Arid Desert Region, Urumqi 830017, China; 3 School of Transportation Engineering, Xinjiang University, Urumqi 830017, China; 4 Xinjiang Institute of Transportation Science and Technology Co., Ltd., Urumqi 830017, China

**Keywords:** trajectory data, risky driving behavior, ELM extreme learning machine, AdaBoost

## Abstract

**Highlights:**

**What are the main findings?**

- Five types of risky driving behaviors were extracted from GPS trajectory data using a threshold method, forming a dedicated dataset.
- The proposed ELM-AdaBoost model achieved 95.8% average recognition accuracy, outperforming conventional classification algorithms.

**What are the implications of the main findings?**

- The high-accuracy model enables more reliable automatic detection of risky driving, supporting proactive traffic safety management.
- This approach can be integrated into real-time monitoring systems to alert drivers and reduce crash risks caused by risky maneuvers.

**Abstract:**

The classification of risky driving behaviors is a key aspect of improving road traffic safety management. A major challenge in this field is that fixed empirical thresholds cannot adequately reflect the operational characteristics of specific vehicle fleets, particularly hazardous chemical transportation vehicles operating under mandatory speed supervision. To address this gap, this study proposes a fleet-specific relative threshold labeling framework based on 90th-percentile (P90) statistics, which adaptively identifies five types of risky driving behaviors—speeding, rapid acceleration, rapid deceleration, sharp turning, and sharp lane changing—from GPS trajectory data. Using this framework, risky driving events were extracted from 481 trip segments and a dedicated risky driving behavior dataset was constructed. To validate the effectiveness of the proposed labeling strategy, an Extreme Learning Machine combined with Adaptive Boosting (ELM-AdaBoost) was employed as the recognition model and compared with commonly used classification algorithms. The results show that the model trained on the P90-labeled dataset achieves an average recognition accuracy of 95.8% for the five behavior categories, outperforming Random Forest, BP neural network, and standalone ELM, thereby confirming that the proposed labeling framework produces a well-separated and learnable dataset.

## 1. Introduction

Road traffic safety remains a major public health and transportation challenge worldwide. According to the World Health Organization (WHO), approximately 1.19 million people die each year as a result of road traffic crashes [1]. In recent years, rapid socioeconomic development and the continuous improvement of residents’ living standards have led to a rapid increase in motor vehicle ownership, while also bringing increasingly serious road traffic safety challenges. According to the latest statistical reports, by the end of 2024, the number of motor vehicles in China had reached 453 million, an increase of 18 million compared with the previous year, and the number of motor vehicle drivers had reached 542 million, an increase of approximately 19 million compared with the previous year [2,3,4]. The growth in motor vehicle ownership has been accompanied by prominent road traffic safety problems. In 2023, more than 250,000 road traffic accidents occurred nationwide, resulting in approximately 310,000 casualties and nearly 1.2 billion yuan in direct property losses [5]. Existing studies indicate that driver behavior accounts for more than 90% of the factors contributing to road traffic accidents [6], among which risky driving behaviors such as speeding and emergency lane changing are particularly prominent. Therefore, the identification and analysis of driver behavior can effectively improve driving safety, reduce the incidence of traffic accidents, and promote the overall improvement of road traffic safety management.

Vehicle GPS trajectory data have been widely used in driving behavior analysis because of their convenient acquisition and continuous spatiotemporal recording capability. Such data usually include vehicle speed, time, latitude, longitude, direction angle, and other information, through which the risky driving behavior of drivers during vehicle operation can be analyzed. Risky driving behavior detection has significant value for traffic agencies in developing effective traffic safety management and intervention strategies. At present, domestic and international studies on risky driving behavior mainly focus on the classification of microscopic driving behaviors. These studies commonly use speed and direction characteristics to analyze behaviors such as rapid acceleration and rapid deceleration during driving [7]. Yang et al. [8] analyzed risky driving behaviors, including speeding, emergency braking, and abnormal stopping of public buses, using real-time GNSS trajectory data. Ma et al. [9] used vehicle kinematic data and driver facial expression data as inputs and constructed a long short-term memory network to recognize five driving behaviors: normal driving, acceleration, deceleration, turning, and lane changing. Boateng et al. [10] aggregated speed, direction, longitude, and latitude data into minute-level segmented data, used singular value decomposition for dimensionality reduction, and finally applied K-means clustering to identify different driving patterns, including harsh braking, harsh acceleration, excessive steering, excessive lane changing, and other behaviors. To quantify the risk level of drivers’ driving behaviors, Zhu Xinglin et al. [11] proposed a real-time identification method for risky driving behaviors. In their study, Lagrange polynomial interpolation was used to preprocess vehicle trajectory data, key motion feature parameters were extracted, and four high-risk driving behaviors, namely sharp turning, rapid acceleration, emergency braking, and speeding, were quantitatively evaluated through an established risk assessment model.

With the development of machine learning and ensemble learning methods, many scholars have attempted to improve the recognition performance of risky driving behavior by constructing more effective classification models. Due to the limited recognition ability of a single algorithm, integrated learning algorithms have been increasingly used for driving behavior recognition and have achieved improved results [12,13,14]. To improve the recognition accuracy and efficiency of risky driving behaviors of key operating vehicles, Zhao et al. [15] constructed a hybrid recognition model integrating time series symbolic analysis and a multiscale convolutional neural network. The model classified abnormal driving behaviors into four types, namely speeding, emergency stopping, temporary stopping, and low-speed driving, according to vehicle type and speed-limit-related characteristic parameters, and constructed an abnormal driving dataset to identify high-risk driving behaviors. Zhang et al. [16] combined a convolutional neural network and a bidirectional gated recurrent unit to analyze extreme acceleration and steering information and construct a driving behavior recognition dataset. The dataset was then input into the CNN-BiGRU model to identify abnormal driving behaviors. In this model, the CNN captures nonlinear relationships from the long-term trend of the sequence, while the BiGRU extracts time-series features from driving parameters for five behaviors: normal driving, rapid acceleration, rapid deceleration, rapid left lane changing, and rapid right lane changing. Chen et al. [17] proposed an effective recognition method based on convolutional neural networks and transfer learning, in which the parameters of a pre-trained CNN model were fine-tuned to identify risky driving behaviors such as acceleration, deceleration, turning, and lane changing [18].

Although existing studies have achieved considerable progress in risky driving behavior recognition, several limitations remain. First, fixed empirical thresholds may not adequately reflect the operational characteristics of specific vehicle types and driving environments, particularly for hazardous chemical transportation vehicles. Second, some recognition methods rely on complex model architectures or multimodal data sources, which may limit their applicability in lightweight GPS-based monitoring scenarios. Third, adaptive behavior-labeling methods for fleet-specific operational environments remain insufficiently explored. To address these limitations, this study focuses on GPS trajectory data from hazardous chemical transportation vehicles and develops a P90-based relative threshold labeling framework to identify risky driving behaviors. Based on the constructed dataset, an ELM-AdaBoost classification model is employed to evaluate the effectiveness of the proposed labeling strategy and compare its performance with commonly used machine learning methods.

Considering that this study focuses on a lightweight GPS-trajectory-feature-based recognition framework, RF, BP, and ELM were selected as representative comparison models because they can be trained using the same manually derived kinematic features as the proposed ELM-AdaBoost model. Deep learning models such as LSTM, CNN-BiGRU, and CNN-based transfer learning generally require sequence-level or high-dimensional feature representations, and their systematic comparison will be further investigated in future work.

The main contributions of this study can be summarized as follows:

(1) A fleet-specific relative threshold labeling framework based on P90 percentile statistics is proposed for hazardous chemical transportation vehicles, enabling adaptive identification of risky driving behaviors from GPS trajectory data.
(2) A risky driving behavior dataset containing five categories of risky behaviors, including speeding, rapid acceleration, rapid deceleration, sharp turning, and sharp lane changing, is constructed using the proposed labeling methodology.
(3) An ELM-AdaBoost classification model is employed to validate the effectiveness of the constructed dataset and is compared with several commonly used machine learning algorithms.

## 2. Materials and Methods

### 2.1. GPS Trajectory Data Preprocessing

The GPS trajectory records used in this study were obtained from the on-board positioning terminals of hazardous chemical transportation vehicles through the transportation management platform of a petroleum company. Each record contains trip segment, vehicle ID, positioning time, speed, longitude, latitude, heading direction, and road-section information. This study focuses on preprocessing existing GPS trajectory records and recognizing risky driving behaviors, rather than designing or evaluating a new sensing device. The exact models of the on-board GPS terminals and video recorders, as well as their nominal positioning accuracy, were not disclosed by the data provider, which is acknowledged as a limitation of this study.

Vehicle trajectory data is a spatio-temporal sequence of information collected in real time through vehicle networking technology, which completely records the dynamic parameter information such as speed, latitude and longitude position, direction, etc., of the vehicle in the operation process [19], and at the same time, it contains static attribute features such as the vehicle ID and vehicle model. This type of data can accurately reflect the vehicle’s motion state, such as acceleration, deceleration, stationary state and its spatio-temporal distribution pattern, providing an important data source for driving behavior analysis and operation state identification [20]. The vehicle trajectory data and on-board video data used in this study were obtained from the transportation vehicles of a petroleum company between May 2023 and November 2023. The raw GPS trajectory dataset contained 268,623 trajectory records from 481 trips made by 57 drivers. Each GPS record included eight fields, trip segment, license plate number, positioning time, speed (km/h), longitude, latitude, heading direction (°), and road-section location, as shown in Table 1. After data preprocessing, 264,139 valid GPS records were retained, and 4484 abnormal records were processed. After HMM-based trajectory correction, most drift anomalies were effectively eliminated. As a result, the residual drift occurrences in the processed dataset are negligible and may not appear in segment-level visualizations. It should be noted that these records were collected only from this petroleum company’s own transportation fleet, so the speed and motion parameters used in the following analysis reflect the operating characteristics of the monitored fleet rather than those of the general traffic stream on the same roads. In addition, because the trips correspond to the round-trip logistics operations of tanker vehicles, the dataset inherently contains both laden (loaded) and unladen (empty) operating conditions; however, the GPS records do not contain an explicit load-state field, so the laden and unladen segments are not separately labelled in the raw data.

All sensitive information is strictly desensitized: the original license plate data is converted to the digital sequence 1 to 57, and the actual geographic location is replaced by the standardized road section number 1 to 366, which ensures both data privacy and integrity of spatial topological relationships, and the data architecture can effectively support the motion state characterization and temporal correlation characteristics required for driving behavior analysis.

Vehicle GPS trajectory data is susceptible to interference by a variety of objective factors during the acquisition process, such as interruption of communication signal transmission, extreme weather, and the impact of building complexes, and this interference will lead to the presence of multiple types of abnormal phenomena in the output raw trajectory data, which must be systematically pre-processed on the raw data to improve the quality of the GPS trajectory sequence data and to eliminate the bias brought about by the data anomalies, in order to ensure the subsequent reliability and integrity of the study of risky driving behavior using GPS trajectory sequence data.

#### 2.1.1. Redundant Data

Redundant data in GPS trajectory data refers to the fact that in the process of vehicle trajectory acquisition, due to the delay in signal transmission, repeated reporting by the equipment or parallel acquisition by multiple systems, etc., the trajectory of the same vehicle appears multiple identical or similar data information at the same time point, and this kind of data redundancy will seriously affect the accuracy of the subsequent analysis, and it is necessary to establish a corresponding processing mechanism.

Using the spatial position consistency detection method for duplicate data identification, by analyzing the spatial and temporal characteristics of adjacent sampling points, the duplicate collection of trajectory sequences are screened, and if the positioning time, direction angle, latitude and longitude coordinates and other information are the same in multiple sampling points, they are judged to be redundant duplicate data, and at this time, only the first trajectory sequence record data in the duplicate data is retained, and the other duplicate values are deleted.

#### 2.1.2. Error Data

There are two types of error data in trajectory data: one is that the value exceeds the reasonable range, such as the speed value exceeds the physical limit value of the vehicle; the other is that the data logic violates the common sense, such as the continuous position jumping within a short period of time, and this kind of problem needs to be dealt with through the setting of the threshold range and the constraints of the logical relationship.

Error data is divided into abnormal latitude and longitude and abnormal speed, the data used in this study has a fixed driving range with clear location boundaries, so the latitude and longitude data that is not within this range will be deleted; during the actual driving process of the vehicle, due to the equipment transmission problem, there will be some data that exceeds the reasonable speed limit, which needs to be screened out and deleted. The maximum speed limit value of the road section where the trajectory data used in this study is 120 km/h, and the maximum value of the speed limit of the hazardous chemical transportation vehicle traveling on the road is 80 km/h [21], and also considering the equipment measurement error and instantaneous acceleration, the speed is set to be 150 km/h as the erroneous speed threshold, and data larger than this threshold are deleted from the speed field.

#### 2.1.3. Missing Data

Due to equipment failure or signal interference and other factors, GPS trajectory data often appear key fields missing phenomenon, including latitude and longitude position information, speed and other motion status information or time stamps. This kind of missing data will lead to broken trajectory or misplaced time, which seriously affects the accuracy of the subsequent analysis.

Since the GPS trajectory data acquisition frequency is the same for each trip, for the neighboring trajectory points whose time interval exceeds the acquisition frequency, the missing data need to be filled in. In this study, linear interpolation is used to fill in the missing data, which is one of the interpolation techniques that are widely used in the field of data processing by establishing the linear relationship between the neighboring valid data points and estimating and reconstructing the missing values, and compared with the complex interpolation method, linear interpolation has the advantages of simple calculation and fast realization, which can effectively maintain the continuity and change trend of data without introducing too much computational complexity. Its core assumption is that the object maintains a linear motion state with constant velocity during the missing time period, so the position of the missing point can be linearly inferred using the known trajectory points. Specifically, as shown in Equations (1) and (2), linear interpolation was performed only for short missing intervals. For trajectory sequences with excessive missing data, the corresponding segments were directly eliminated to prevent adverse effects on subsequent risky driving behavior analysis.

(1) $$x=x_{1}+\frac{\left(t-t_{1}\right)}{\left(t_{2}-t_{1}\right)}\times (x_{2}-x_{1})$$

(2) $$y=y_{1}+\frac{\left(t-t_{1}\right)}{\left(t_{2}-t_{1}\right)}\times (y_{2}-y_{1})$$

In the formula, it is the timestamp of the missing point; *x*,*y* is the interpolated value of the longitude and latitude of the missing point; *t*_1_ and *t*_2_ represent the timestamps of two adjacent trajectory points with missing values; *x*_1_ and *x*_2_ are the longitude coordinates of two adjacent trajectory points with missing values; *y*_1_ and *y*_2_ are the latitude coordinates of two adjacent trajectory points with missing values.

To reduce the influence of interpolation-induced smoothing, linear interpolation was applied only to short missing intervals to maintain basic trajectory continuity, whereas trajectory segments with excessively long missing intervals were discarded rather than interpolated. In total, approximately 2113 GPS records (about 0.8% of the 264,139 valid records) were reconstructed by interpolation. Each interpolated record was assigned a binary flag during preprocessing. During candidate-event extraction, every event whose calculation window—the set of adjacent GPS records used to compute acceleration and angular velocity—contained at least one flagged record was discarded before label generation. Under this rule, 149 candidate events were removed, so that no retained labeled event overlapped with interpolated records, and all final labels were generated exclusively from non-interpolated trajectory data.

#### 2.1.4. Data Drift

Data drift refers to the abnormal data phenomenon that the GPS positioning point is significantly offset from the actual position, and this kind of data problem will significantly affect the accuracy of the trajectory analysis and the consistency of the trajectory.

For GPS trajectory drift data, this study adopts the Hidden Markov Model (HMM) [22] to correct the position of the drifting trajectory points. By analyzing the probabilistic relationship between the implied state of the real road position of the trajectory point and the observed state of the drift point, the drift trajectory point is matched to the correct road.

**Figure 1:**
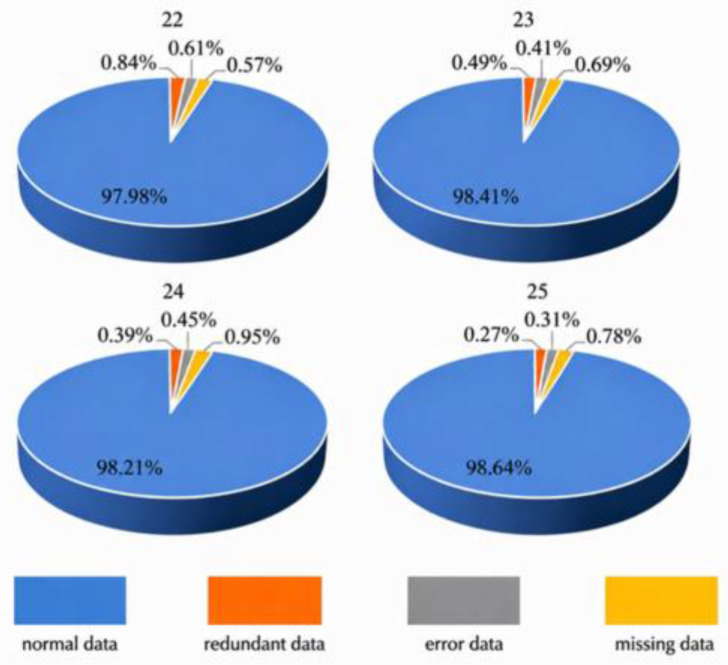
Representative visualization of trajectory data quality across four trip segments, including valid, redundant, error, missing, and drift data: The figure illustrates typical data-quality patterns, while overall statistics are based on the full preprocessed dataset. Drift data may be visually negligible in some segments due to its extremely low proportion after HMM-based correction.

After data preprocessing, 264,139 valid records were obtained, and 4484 abnormal records were processed. The percentage of valid data was approximately 98%, while problematic data accounted for approximately 2%. Therefore, the overall data quality evaluation in this study is based on the preprocessing statistics of the complete dataset. Figure 1 provides a representative visualization of four trip segments only for illustrating typical data-quality patterns, including valid data, redundant data, error data, missing data, and drift data.

The preprocessing procedures in this section, including redundant-data removal, error-data screening, missing-data interpolation, and HMM-based drift correction, are intended to improve the usability and consistency of the collected GPS trajectory records. They should therefore be interpreted as trajectory-data processing steps rather than as an evaluation or improvement of GPS sensor hardware performance.

### 2.2. Classification of Risky Driving Behavior

In this study, the 90th percentile (P90) was selected as the relative threshold for identifying risky driving behaviors. P90 represents the upper tail of the observed driving-behavior distribution while retaining sufficient samples for model training. Compared with P85, P90 is less likely to label moderate but acceptable driving fluctuations as risky behaviors; compared with P95, it avoids generating too few risky samples, which could aggravate class imbalance. Therefore, P90 provides a practical balance between risk sensitivity and sample availability for the studied hazardous chemical transportation fleet. It should be noted that the P90 threshold is a fleet-specific relative criterion rather than a universal safety standard.

To examine whether the selected percentile substantially affects label construction and model performance, a sensitivity analysis was conducted using P85, P90, and P95 as candidate thresholds. As shown in Table 2, the number of samples in the constructed dataset decreased from 9247 at P85 to 6825 at P95, indicating that higher percentile thresholds identify more conservative and extreme risky driving behaviors. The ELM-AdaBoost accuracy remained stable across the three settings, ranging from 94.6% to 95.8%. The P90 threshold was selected for final dataset construction. Under this setting, a total of 8016 driving samples were generated for the final classification dataset, including both normal and risky driving behaviors; the detailed class distribution is presented in Section 2.2.5. Therefore, P90 was adopted in this study because it provides a balance between risk sensitivity, sample availability, and recognition performance.

The physical interpretability of the proposed thresholds was also assessed by comparing the obtained values with driving-behavior indicators reported in previous studies. Osafune et al. reported that acceleration exceeding 2.4 m/s^2^ and deceleration exceeding 1.4 m/s^2^ could be used to distinguish risky drivers from safe drivers [23]. In this study, the rapid acceleration threshold (2.22 m/s^2^) and rapid deceleration threshold (−1.83 m/s^2^) are of a comparable magnitude to these reported values. The sharp-turning threshold (15.35°/s) and sharp lane-changing threshold (8.46°/s) also correspond to the upper tail of the observed angular-velocity distribution. To enable an independent observational check, 120 threshold-identified events were randomly sampled for video cross-validation, stratified across the five behavior categories (24 events per category) so that every behavior type was represented. These events were cross-checked against the corresponding onboard video records, and 89% of the inspected events were judged to be consistent with the labeled risky maneuver based on observable driving behavior in the videos. In addition, 120 threshold-identified events were cross-checked with the corresponding onboard video records, and 89% of the inspected events were judged to be consistent with the labeled risky maneuver based on observable driving maneuvers in the videos. These results indicate that the P90-based thresholds are physically interpretable and can provide a reasonable basis for subsequent risky driving behavior recognition.

By analyzing the two key features of speed and heading direction in the trajectory data, risky driving behavior indicators can be extracted from GPS trajectories. The risky behaviors related to speed include speeding, rapid acceleration, and rapid deceleration, while those related to heading direction include sharp turning and sharp lane changing. Based on the characteristics and hazards of these five types of risky driving behaviors, a percentile-based threshold method was used to extract risky driving events during vehicle operation, and a risky driving behavior dataset was constructed from the GPS trajectory data.

#### 2.2.1. Speeding

Speeding driving behavior refers to the phenomenon that the actual driving speed of a vehicle during driving exceeds the legal speed limit threshold of a specific road section at the corresponding time. Speeding will significantly increase the risk of traffic accidents, excessive speed will significantly shorten the effective reaction time of the driver, and it is difficult to take risk avoidance measures in a timely manner in case of emergencies, and the speed exceeding the road design standard will destroy the stability of vehicle handling, which is very likely to lead to side-slipping, rear-end collision, or even overturning and other out-of-control conditions. Previous studies have demonstrated that speeding behavior is strongly associated with increased crash risk and injury severity, highlighting the importance of speed management for traffic safety [24].

**Figure 2:**
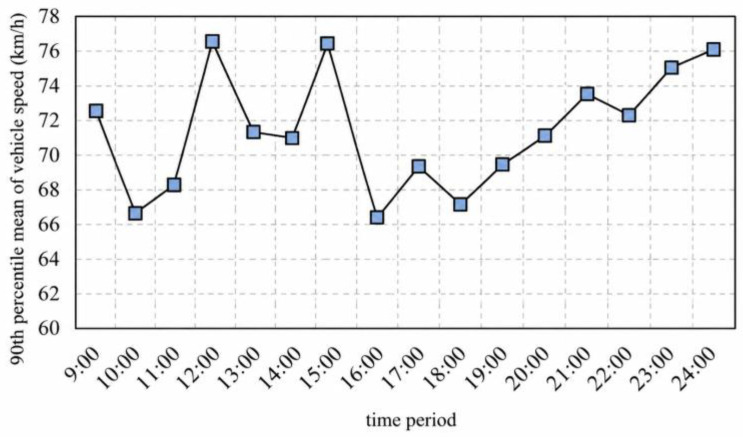
The 90th percentile mean value of vehicle speed throughout the day for the road section numbered 6: This figure shows the hourly 90th-percentile speed values of vehicles traveling on road section No. 6. The P90 value was calculated based on the speed distribution of vehicles passing through this road section during different time periods. The average P90 speed was 71.36 km/h, which was used as the relative speeding threshold for this road section. Since no vehicles traveled on this road section from 01:00 to 08:00, no speed statistics were calculated for this time period.

In China, the posted speed limit of the road sections covered by this study is 120 km/h, whereas the maximum speed permitted for hazardous chemical transportation vehicles on these roads is 80 km/h according to the relevant safety management regulations [21]. Because this monitored fleet is subject to mandatory on-board speed supervision, the vehicles seldom exceed the 80 km/h regulatory cap, so the number of strictly illegal speeding events is too small to support reliable model training. Therefore, instead of judging speeding solely against the fixed legal limit, this paper additionally introduces a “relative speeding” criterion that identifies vehicles travelling markedly faster than their peers under identical road and time conditions. It should be emphasized that the speed samples used to derive this criterion are drawn only from the studied transportation fleet rather than from all vehicles on the road; the resulting threshold therefore reflects the speed distribution of this specific fleet and is interpreted as a peer-referenced (relative) indicator rather than an absolute, legally defined speed limit. Specifically, for each of the 366 road sections, the speeds of all monitored fleet vehicles passing through the section are aggregated using a one-hour statistical interval and sorted in ascending order, and the 90th-percentile mean speed (P90) is taken as the relative speeding threshold of that section. Taking road section No. 6 as an example, the hourly P90 speed throughout the day is shown in Figure 2; the daily average P90 speed of this section is 71.36 km/h, which is adopted as its relative speeding threshold. Notably, this relative threshold (71.36 km/h) lies below the 80 km/h regulatory cap, indicating that the relative criterion is more sensitive than the legal limit and can flag aggressive speed behavior that a fixed-threshold approach would otherwise miss. Since no vehicles travelled on this section between 01:00 and 08:00, no speed statistics were computed for this period.

#### 2.2.2. Rapid Acceleration and Deceleration

Rapid acceleration and deceleration behavior refers to driving operations in which a vehicle suddenly accelerates or decelerates in a short period of time, usually manifested as a drastic change in the gas pedal or brake. This kind of behavior may be triggered by the driver’s subjective intention, such as robbing, road rage or sudden obstacles, sudden changes in signal lights and other objective conditions. The parameters of the characteristics of the rapid acceleration and deceleration operation are the core elements for measuring the safety of the driving behavior, which occupies a key position in the risk warning system of the intelligent traffic control system. Although this type of driving behavior is not included in the current traffic laws and regulations of the explicit punishment category, but often accompanied by high-risk driving mode, not only significantly increase the risk of traffic accidents, but also lead to vehicle loss and fuel waste.

From the perspective of behavioral characteristics, rapid acceleration is mainly manifested as a sudden increase in speed within a short period of time, and occurs in two typical scenarios: first, urban congested roads, due to frequent starts and stops and short intersections that lead drivers to step on the gas pedal; and second, open roads in the suburbs, due to the scarcity of traffic and insufficient supervision that induces aggressive driving.

This type of behavior exacerbates the risk of vehicle rear-end collisions and leads to abnormal wear and tear on the engine, braking system and tires.

Rapid deceleration behavior, on the other hand, is mainly manifested as a sharp drop in speed, and the main reasons are divided into three aspects: first, emergency braking when the following distance is insufficient; second, sudden changes in the road line; and third, the risk of losing control under adverse weather conditions.

Especially at high speeds, rapid deceleration is very likely to cause multi-vehicle rear-end accidents, and may also cause vehicle skidding under low adhesion coefficient road conditions.

At present, thresholds for rapid acceleration and deceleration are commonly determined using empirical values. In addition, recent studies have investigated acceleration- and deceleration-related driving behavior indicators under different operational scenarios to improve driving safety assessment and aggressive driving identification [25,26]. In this study, when determining the threshold of rapid acceleration and deceleration, we take into account that the injuries caused by acceleration of the same size may be different for different types of vehicles in the process of traveling, such as hazardous chemical transportation vehicles compared to ordinary vehicles, the injuries caused by excessive acceleration may be higher. Therefore, similar to the method of defining the speeding threshold, with “relative rapid acceleration and deceleration” as the research objective, this study aims at the special vehicle of hazardous chemical transportation vehicles, traverses the speed information of all trajectory points of all monitored fleet vehicles in all travel segments, calculates the inter-area acceleration of the neighboring trajectory points, divides the positive acceleration and negative acceleration, and ranks the absolute value of the two acceleration types in the order of smallest to largest. The absolute values of the two types of acceleration are sorted from small to large, and the average value of acceleration P90 is extracted as the threshold of rapid acceleration and deceleration, and the final calculated thresholds of rapid acceleration and rapid deceleration are 2.22 m/s^2^ and −1.83 m/s^2^, respectively.

#### 2.2.3. Sharp Turning

Sharp turning behavior refers to the risky driving behavior that the driver fails to reasonably control the speed and steering angle during the turning process, and completes the steering operation with too high lateral acceleration. This behavior is common at intersections and curves, often accompanied by failure to decelerate in advance, turning too quickly and other characteristics. The main hazards of sharp turning are divided into three categories: first, it is very easy to cause the vehicle to skid or out of control, especially on slippery roads or the vehicle center of gravity is higher; second, it is a substantial increase in the risk of rollover, SUVs, trucks and other vehicles with high centers of gravity is particularly dangerous; third, it is a serious impact on the comfort of the ride, which may result in passenger seasickness or injuries.

Different vehicle types have different sensitivities to sharp turns, taking “relative sharp turns” as the research objective, traversing all the samples in which the change in direction angle is greater than 90 degrees in all travel segments, calculating their angular velocities according to Equation (3), sorting the angular velocities according to the order of smallest to largest, and extracting the mean value of angular velocity P90 as the threshold for sharp turns. The sharp turn threshold is 15.35 degrees/second.

(3) $$w_{i(1)}=\left|\frac{c_{i+1}-c_{i}}{t_{i}}\right|$$

#### 2.2.4. Sharp Lane Change

Sharp lane change is a risky driving behavior in which the driver suddenly and drastically changes lanes without fully observing the surrounding road conditions or maintaining a safe distance. This behavior is usually manifested in the use of turn signals is not standardized, changing lanes too large an angle or changing lanes too fast, is very easy to damage the normal traffic flow order and cause serious safety hazards, the harm of the behavior of the sharp lane change is mainly manifested in three aspects: First, greatly increase the risk of lateral collision, especially at high speeds can easily lead to loss of control of the vehicle; second, forcing other vehicles to avoid emergency, may trigger a chain-reaction type of accidents; Third, to Exacerbate traffic congestion, frequent sharp lane changes will disrupt the continuity of traffic flow.

Iterate through all travel segments and identify samples with heading-angle changes greater than 6°. To distinguish sharp lane-changing behavior from sharp turning behavior, only samples with heading-angle changes between 6° and 90° were extracted. The angular velocity of these trajectory segments was calculated using the same angular velocity equation (Equation (3)) as that used for sharp turning. The difference between the two behaviors lies in the heading-angle variation criterion rather than the calculation method. Specifically, sharp turning events correspond to heading-angle changes greater than 90°, whereas sharp lane-changing events correspond to heading-angle changes between 6° and 90°. The calculated angular velocities were then ranked from smallest to largest, and the average P90 angular velocity was adopted as the threshold for sharp lane-changing behavior. The final threshold for sharp lane changing was determined to be 8.46°/s.

#### 2.2.5. Sample Construction and Class Distribution

Based on the threshold-based event extraction rules described above, candidate driving-behavior events were extracted from the preprocessed GPS trajectory records. All GPS records within each trip segment were first sorted by positioning time. Candidate driving-behavior events were then extracted from adjacent valid GPS records according to the threshold rules described in Section 2.2.1, Section 2.2.2, Section 2.2.3 and Section 2.2.4, rather than treating an entire trip as one sample. Acceleration and deceleration were calculated from the speed differences between adjacent GPS records, while angular velocity was calculated according to Equation (3). For ELM-based classification, the input vector of each sample consisted of vehicle speed, acceleration/deceleration, heading-angle change, angular velocity, road-section-specific P90 speed threshold, and relative speeding margin, as summarized in Table 3. If a candidate event satisfied more than one risky-driving criterion, the final label was assigned according to the largest normalized threshold exceedance to avoid multi-label ambiguity. In the extracted candidate set, such multi-criterion events accounted for approximately 7%, with speeding together with rapid acceleration being the most frequent co-occurrence. Among them, about 1.5% of all candidate events had two leading normalized exceedances differing by less than 5% and were therefore flagged for ambiguity inspection. After further inspection, 0.9% of candidate events remained genuinely unresolvable and were excluded from the final dataset. This small exclusion proportion indicates negligible information loss arising from multi-label conflicts. Regarding the relative speeding margin (the difference between vehicle speed and the road-section P90 threshold), this feature is computed for every sample regardless of its behavior category and is retained as a signed value. For non-speeding behaviors such as rapid deceleration or sharp turning, the margin is typically zero or negative, indicating that the vehicle was travelling at or below its fleet-referenced speed level during the event. Rather than being a source of bias, this signed feature provides discriminative contextual information: it allows the classifier to distinguish, for example, a directional maneuver performed at a normal speed from one performed while travelling faster than peers. The feature was therefore not zeroed or removed for non-speeding classes; instead, all input features were normalized prior to training (Section 2.4) so that the speeding margin and the other kinematic features share a comparable numerical scale across all categories.

The final classification dataset contained 8016 samples, including 3000 normal-driving samples and 5016 risky-driving samples. The risky-driving samples consisted of five behavior categories: speeding, rapid acceleration, rapid deceleration, sharp turning, and sharp lane changing. The detailed class distribution is shown in Table 4.

### 2.3. ELM-AdaBoost-Based Risky Driving Behavior Recognition

The threshold-based method described in Section 2.2 and the ELM-AdaBoost model serve different purposes in this study. The threshold method is used to identify risky driving events from GPS trajectory data and generate behavior labels for dataset construction. Based on the labeled dataset, the ELM-AdaBoost model is trained to recognize risky driving behaviors from trajectory features. Unlike the threshold-based labeling procedure, the trained classifier can directly predict behavior categories from individual samples without recalculating fleet-level statistical thresholds. In this study, ELM-AdaBoost is employed as the proposed multi-class recognition model, while RF, BP, and ELM are used as baseline models for performance comparison.

#### 2.3.1. Extreme Learning Machine (ELM)

**Figure 3:**
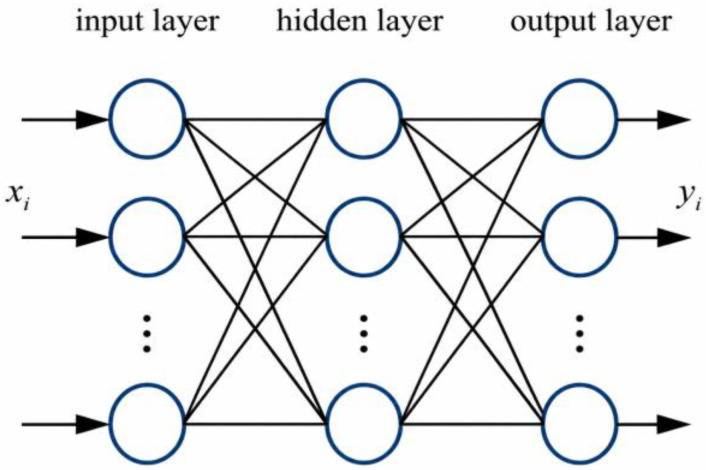
ELM network topology: This figure illustrates the basic structure of the Extreme Learning Machine model, which consists of an input layer, a hidden layer, and an output layer. The input layer receives trajectory-related driving behavior features, the hidden layer performs nonlinear feature mapping through randomly assigned input weights and biases, and the output layer generates the final classification result. Compared with traditional neural networks, ELM can determine the output weights analytically, which improves training efficiency and makes it suitable for lightweight risky driving behavior recognition tasks.

Extreme Learning Machine (ELM) [27] as a supervised learning algorithm, the core point of which lies in the design of a single hidden layer feed-forward neural network using a random initialization strategy. The algorithm contains three basic layers in terms of structural composition: input layer, hidden layer and output layer, as shown in Figure 3.

Compared with traditional neural networks, ELM has significant computational efficiency advantages, the connection weights and bias terms of the hidden layer are randomly generated by probability distribution and do not require iterative optimization, and the model training can be completed by solving the weight matrix of the output layer by analytical method. ELM determines the optimal solution by the optimization criterion of minimizing the output error. During model training, the hidden layer input weight matrix and neuron bias are randomly assigned according to a specific probability distribution, and then the output weights are directly calculated by solving a system of linear equations. When the loss function reaches the global minimum, the optimal model parameter configuration is obtained. This unique training mechanism makes ELM maintain good generalization performance while significantly improving the model training speed.

The specific calculation steps of ELM model are as follows:

(1) Assume that the total number of samples is *N* and the dataset S = [*X*,*T*], the input data is:
  (4) $$X={\left[\begin{array}{cccc} X^{\left(1\right)} & X^{\left(2\right)} & \cdots & X^{\left(N\right)} \end{array}\right]}_{n\times N}$$
  (5) $$X^{\left(i\right)}={\left(\begin{array}{cccc} x_{1} & x_{2} & \cdots & x_{n} \end{array}\right)}^{T}$$
  where is the input feature vector dimension.
(2) The output label is:
  (6) $$T={\left[\begin{array}{cccc} T^{\left(1\right)} & T^{\left(2\right)} & \cdots & T^{\left(N\right)} \end{array}\right]}_{N\times m}$$
  (7) $$T^{\left(i\right)}={\left(\begin{array}{cccc} t_{1} & t_{2} & \cdots & t_{m} \end{array}\right)}^{T}$$
  where is the output vector dimension.
(3) The hidden layer input weights *W*:
  (8) $$W={{\left[\begin{array}{cccc} W_{1} & W_{2} & \cdots & W_{L} \end{array}\right]}^{T}}_{L\times n}$$
  (9) $$W_{j}=(\begin{array}{cccc} w_{j1} & w_{j2} & \cdots & w_{jn}{)}^{T} \end{array}$$
  where *L* is the number of nodes in the hidden layer.
(4) Hidden layer input bias *b*:
  (10) $$b={\left(\begin{array}{cccc} b_{1} & b_{2} & \cdots & b_{L} \end{array}\right)}^{T}$$
(5) Hidden layer output weights *β*:
  (11) $$\beta ={{\left[\begin{array}{cccc} \beta_{1} & \beta_{2} & \cdots & \beta_{L} \end{array}\right]}^{T}}_{L\times m}$$
  (12) $$\beta_{k}=(\begin{array}{cccc} \beta_{k1} & \beta_{k2} & \cdots & \beta_{km}{)}^{T} \end{array}$$

**Figure 4:**
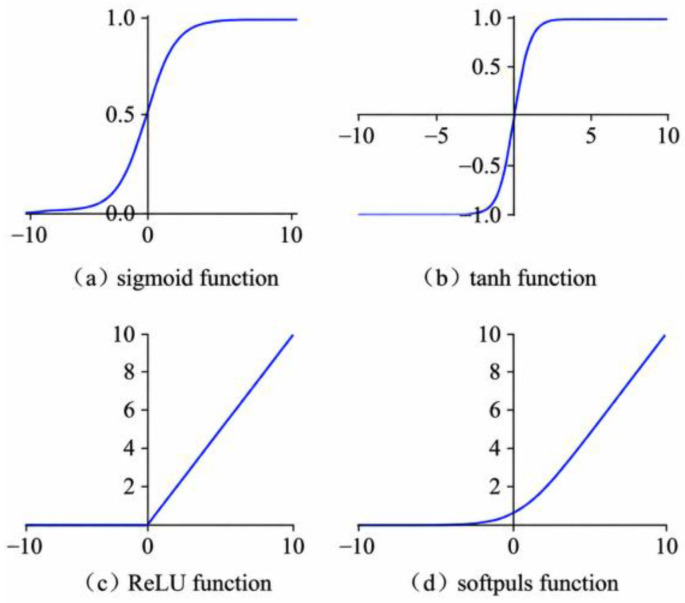
Four commonly used activation function curves: This figure compares four typical activation functions, including the Sigmoid function, Tanh function, ReLU function, and Softplus function. These activation functions provide nonlinear mapping capabilities for neural network models and influence the feature representation ability of the hidden layer. In this study, the Sigmoid function was selected as the activation function of the ELM hidden layer because it can map input features into a bounded nonlinear space and support stable model training for risky driving behavior recognition.

In the feed-forward neural network architecture in the field of deep learning, the activation function, as the core component of the nonlinear transformation, is chosen to directly affect the expressive ability of the model, and there are four types of mainstream activation functions commonly used at present, i.e., the S-type (*sigmoid*) function, the hyperbolic tangent (*tanh*) function, the linear rectification (*ReLU*) function, and the flexible tangent (*softpuls*) function, and the plotted image is shown in Figure 4. In order to improve the ability of the extreme learning machine model to respond to the features of the input data, and then optimize its training performance, this study adopts the *sigmoid* function as the nonlinear activation unit of the implicit layer.

The mathematical expressions of the functions *sigmoid* are defined as follows:

(13) $$g(x)=\frac{1}{1+e^{-x}}$$

A nonlinear processing mechanism is added to the activation function:

(14) $$H_{j}^{\left(i\right)}=g(W_{j}X^{\left(i\right)}+b_{j})=\frac{1}{1+e^{-(W_{j}X^{\left(i\right)}+b_{j})}}$$

Represent the hidden layer with *H* matrix as follows:

(15) $$H(W,b,X)={\left[\begin{array}{ccc} g(W_{1}X^{\left(1\right)}+b_{1}) & \cdots & g(W_{L}X^{\left(1\right)}+b_{L})\\ \vdots & \ddots & \vdots \\ g(W_{1}X^{\left(N\right)}+b_{1}) & \cdots & g(W_{L}X^{\left(N\right)}+b_{L}) \end{array}\right]}_{N\times L}$$

Output the sample:

(16) $$H^{\left(i\right)}\beta =O^{\left(i\right)},i=1,\ldots ,N$$

In the optimization process of the Extreme Learning Machine model, the core objective is to improve the model accuracy by minimizing the difference between the predicted value and the true value, so that the error between the network output and the sample labels reaches the global minimum, thus achieving the fit to the training data and constructing the loss function expression:

(17) $${\sum_{i=1}^{N}\left\|O^{\left(i\right)}-T^{\left(i\right)}\right\|}_{m\times N}=0$$

#### 2.3.2. AdaBoost

**Figure 5:**
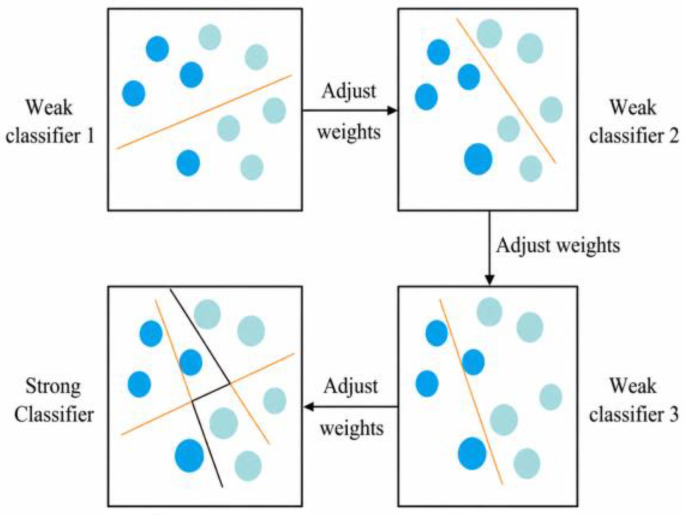
AdaBoost combined strong classifier process: This figure illustrates the basic principle of the AdaBoost ensemble learning mechanism. In the AdaBoost framework, multiple weak classifiers are trained sequentially. During each iteration, the weights of misclassified samples are increased, while the weights of correctly classified samples are decreased. As a result, subsequent weak classifiers pay more attention to samples that are difficult to classify. Finally, all weak classifiers are combined through a weighted voting strategy to form a strong classifier, thereby improving the overall recognition performance of the model. In each panel, the blue and orange circles represent training samples belonging to two different classes, and the size of each circle is proportional to its sample weight; misclassified samples are assigned larger weights and are therefore drawn larger in the subsequent iteration. The green line in each weak-classifier panel denotes the decision boundary learned by that weak classifier, and the strong-classifier panel shows the combined decision boundaries obtained through the weighted voting of the three weak classifiers.

AdaBoost [28] (Adaptive Boosting) is a classical integrated learning algorithm that iteratively trains multiple weak classifiers and assigns different weights to them, and finally combines them into a strong classifier. The core idea is in each iteration, the system dynamically adjusts the sample weight distribution, focusing on improving the weight coefficient of the misclassified samples, so that the subsequent weak classifiers pay more attention to the samples that are difficult to classify, and at the same time, the corresponding weights are assigned according to the accuracy of the classifier, and finally the decision is made through the weighted voting mechanism, as shown in Figure 5. The algorithm has the ability to adaptively adjust the sample distribution, which can effectively improve the model performance, and is especially suitable for dealing with complex boundaries and category imbalance in classification problems.

**Figure 6:**
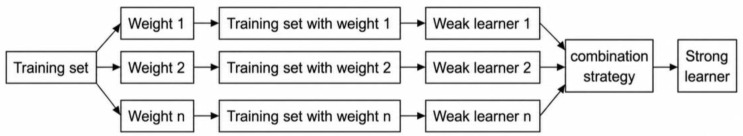
Flow of AdaBoost classification algorithm: This figure presents the iterative training process of the AdaBoost algorithm when ELM is used as the base classifier. After the training sample set is input, all samples are assigned initial weights. In each iteration, an ELM classifier is trained according to the current sample weight distribution, and the classification error is calculated. The sample weights are then updated based on the classification results, so that misclassified samples are assigned larger weights and receive more attention in the next training round. When the preset maximum number of iterations is reached, the trained ELM weak classifiers are combined through weighted voting to generate the final ELM-AdaBoost classifier for risky driving behavior recognition.

The process of AdaBoost classification algorithm is shown in Figure 6, and the specific steps are as follows:

(1) In the initial stage of the algorithm, the uniform distribution assumption is used to assign the same initial weight value to all the training samples to achieve the initialization of the sample weight settings;
(2) Train a weak classifier according to the current sample weights, and update the assigned weights according to the classification error rate of this classifier;
(3) Weighted combination of all trained weak classifiers according to the weights, and the combined strong classifiers make the final decision by weighted voting.

#### 2.3.3. Model of ELM-AdaBoost Algorithm

**Figure 7:**
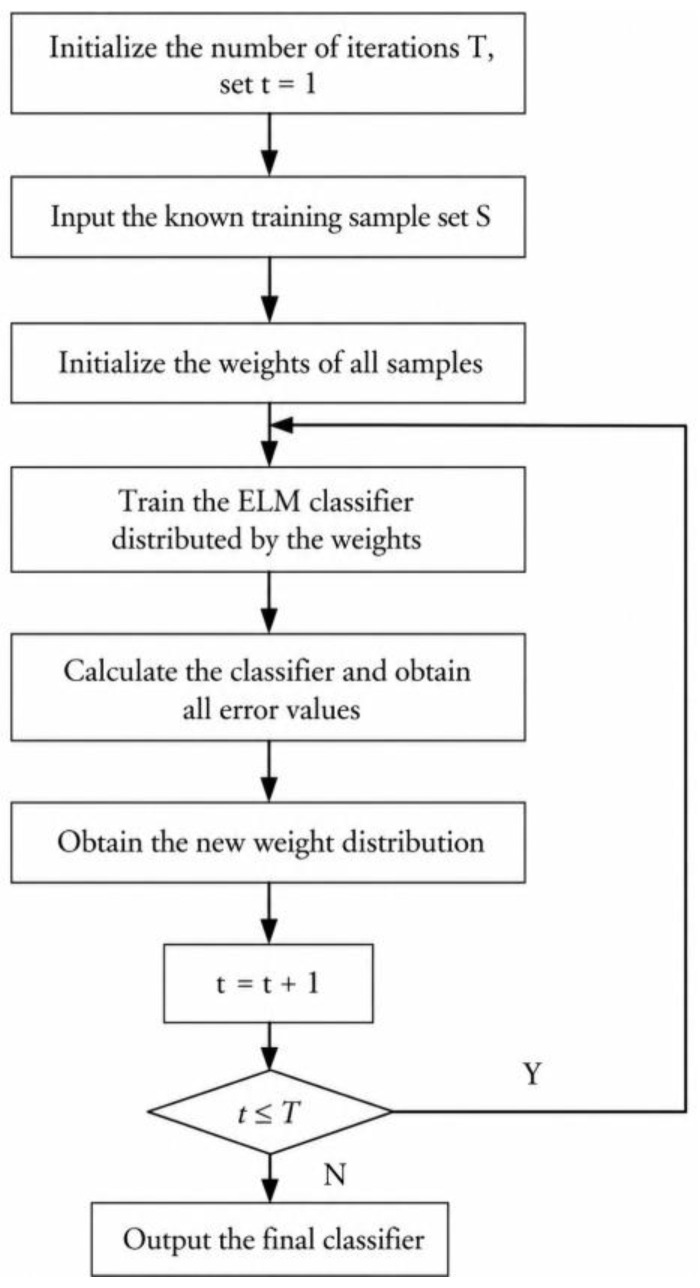
Flowchart of the ELM-AdaBoost model: This figure illustrates the training process of the proposed ELM-AdaBoost model. First, the number of iterations T is initialized and the training sample set S is input. The weights of all samples are then initialized. In each iteration, an ELM classifier is trained according to the current sample weight distribution, and the classification errors are calculated. Based on the error results, the sample weights are updated so that misclassified samples receive greater attention in the next iteration. This process is repeated until the maximum number of iterations is reached. Finally, multiple ELM weak classifiers are combined through the AdaBoost mechanism to obtain the final strong classifier for risky driving behavior recognition.

ELM-AdaBoost is a combination of ELM Extreme Learning Machine and AdaBoost method, which combines the advantages of the two, overcoming the problem of the traditional AdaBoost algorithm’s learning efficiency decreasing due to the growth of the number of samples in the learning process. The model adopts ELM as the basic learning unit, and its unique stochastic parameter initialization strategy and analytic solving method ensure the rapidity of model training; at the same time, it introduces the sequence enhancement mechanism of AdaBoost, which effectively improves the generalization performance of the overall model by dynamically adjusting the distribution of the sample weight and the combination coefficients of the classifier. The flow of the ELM-AdaBoost model is shown in Figure 7, and the single hidden layer feed-forward structure of the ELM module is shown in Figure 7. The single hidden layer feed-forward structure of ELM module realizes feature nonlinear transformation by randomly determining the hidden layer parameters, while the AdaBoost component gradually focuses on the difficult-to-classify samples through the iterative optimization process, and ultimately optimally combines the outputs of multiple weak classifiers in a linear combination, which significantly improves the classification accuracy while maintaining the computational efficiency, and is especially suitable for the complex pattern recognition task under large-scale datasets.

The flow of risky driving behavior recognition using ELM-AdaBoost is as follows:

(1) Initialization of the algorithm: Set the maximum number of iterations T of the AdaBoost algorithm and set the initial weights of the training samples to be uniformly distributed.
(2) Training of the base classifier: Train an ELM classifier based on the current sample weight distribution. The training process of ELM includes randomly generating the hidden layer weights and thresholds, and calculating the output layer weights by the least squares method.
(3) Determination of basic classifier weights: Based on the classification error of the current ELM classifier on weighted samples, calculate the weights of this classifier, the smaller the error, the larger the weight of the classifier.
(4) Adjustment of training sample weights: Dynamically adjust the sample weight distribution according to the prediction results of the current classifier, raise the weight value of misclassified samples, while lowering the weight value of correctly classified samples.
(5) Obtain the final classification function: after completing T iterations, each ELM-based classifier and its corresponding weight coefficients are linearly combined to form the final strong classification decision function.

### 2.4. Experimental Settings and Hyperparameter Configuration

To improve the reproducibility and transparency of the model comparison, the hyperparameter settings of the proposed ELM-AdaBoost model and all baseline models are summarized in Table 5. All models were trained and tested using the same risky driving behavior dataset and the same input feature set. Before model training, continuous variables were normalized to reduce the influence of different numerical scales among speed, acceleration, heading-angle change, and angular velocity. The hyperparameters were determined through preliminary validation experiments and then kept fixed for all comparative tests. To ensure a fair comparison, ELM and BP were configured with the same number of hidden neurons. For the proposed ELM-AdaBoost model, the number of weak classifiers was set to 20 to balance recognition accuracy and computational efficiency. Since the recognition task involves five risky driving behavior categories, the SAMME multi-class AdaBoost strategy was adopted.

As shown in Table 5, the same hidden-layer size was used for ELM, ELM-AdaBoost, and BP to ensure comparability among neural-network-based models. The ELM output weights were calculated using a ridge-regularized Moore–Penrose solution to improve numerical stability. RF was configured with 100 decision trees and a maximum depth of 10 to reduce overfitting while maintaining sufficient classification capability. BP was trained using the Adam optimizer with a cross-entropy loss function and a softmax output layer for multi-class classification. These settings provide a transparent basis for reproducing the reported model comparison results. The preliminary validation experiments used to determine the hyperparameter values were conducted using a subset of the training data separated before the final 10-fold cross-validation splits. The validation criterion was the mean classification accuracy on the held-out validation subset. The final test folds were not accessed during this hyperparameter search, ensuring that the reported cross-validation results are free from data leakage.

All experiments were implemented in Python 3.9 on a Windows 10 64-bit operating system (Microsoft Corporation, Redmond, WA, USA). The experiments were run on a desktop computer equipped with an AMD Ryzen 9 5950X 16-Core Processor CPU (Advanced Micro Devices, Inc., Santa Clara, CA, USA) and 32 GB RAM, without GPU acceleration. The classification models were implemented using scikit-learn 1.3.2 and NumPy 1.24.4. The runtime results reported in Section 3 were obtained under the same software and hardware environment for all compared models to ensure a fair and reproducible comparison.

## 3. Results

To comprehensively evaluate the recognition performance of different models, this study uses Accuracy, Precision, Recall, and F1-score as evaluation metrics. Accuracy reflects the overall proportion of correctly classified samples, while Precision indicates the proportion of correctly identified risky driving samples among all samples predicted as that category. Recall measures the ability of the model to detect actual risky driving samples, and F1-score provides a balanced evaluation by considering both Precision and Recall. Compared with Accuracy alone, these metrics provide a more comprehensive assessment of model performance, especially when the sample distribution among different driving behavior categories is imbalanced.

In this section, based on the proposed threshold decision criterion of risky driving behavior, five types of risky driving behaviors are extracted, and five types of risky driving behavior events are labeled from the trajectory features, which finally form a dataset containing speeding, rapid acceleration, rapid deceleration, rapid turn, and rapid lane change, and establish a multi-label classification dataset of risky driving behaviors.

Taking 481 GPS trips of hazardous materials transportation vehicles as the study dataset, the proposed ELM-AdaBoost model was employed to directly perform multi-class recognition of risky driving behaviors. The recognition results were compared with those of RF, BP, and ELM baseline models to evaluate the effectiveness of the proposed approach.

Using the 8016 samples constructed from the GPS trajectory dataset, model performance was evaluated using stratified 10-fold cross-validation. Stratified sampling was adopted to preserve the class distribution of risky driving behaviors in each fold. Considering that ELM employs randomly initialized hidden-layer weights and biases, the mean accuracy and standard deviation across the ten folds were calculated to evaluate model stability and robustness. To mitigate potential bias arising from differences in class frequencies, the same stratified cross-validation setting was applied to all compared models, including RF, BP, ELM, and ELM-AdaBoost.

**Figure 8:**
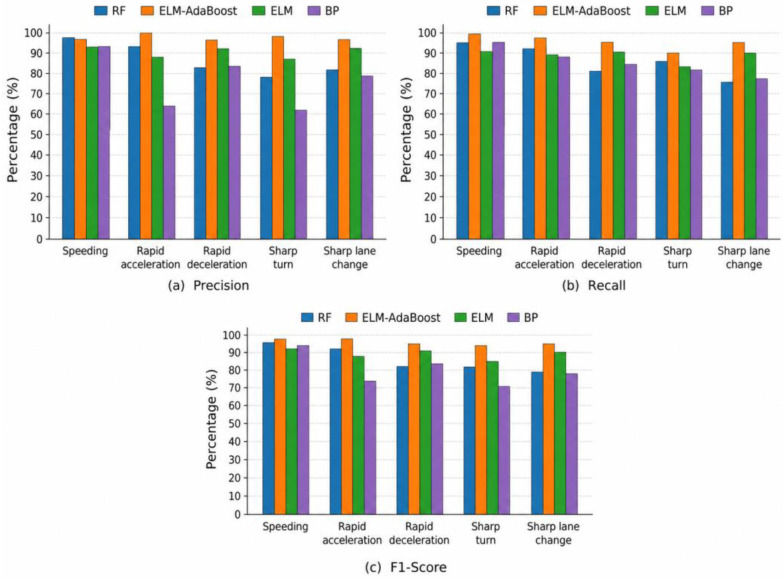
Comparison of classification performance among different models. The Precision, Recall, and F1-score of RF, ELM-AdaBoost, ELM, and BP are compared across five risky driving behavior categories, including speeding, rapid acceleration, rapid deceleration, sharp turning, and sharp lane changing. The results show that ELM-AdaBoost achieves more consistent performance across different categories, suggesting that the AdaBoost ensemble strategy enhances the robustness of ELM-based risky driving behavior recognition.

The recognition results of the four models are shown in Figure 8, and the specific information of each type of evaluation parameter is shown in Table 6, from which it can be concluded that the average recognition accuracy of the ELM-AdaBoost model proposed in this paper is 95.8%, which has a better recognition effect compared with other models.

As shown in Table 6, the proposed ELM-AdaBoost model achieves the best overall recognition performance among the four compared models. Its mean accuracy reaches 95.8 ± 0.7%, indicating that the model maintains high recognition accuracy across different cross-validation folds. In addition, the Precision, Recall, and F1-score of ELM-AdaBoost are higher than those of RF, BP, and the single ELM model in most behavior categories, suggesting that the proposed ensemble model not only improves the overall classification accuracy but also enhances the stability of risky driving behavior recognition. Compared with the single ELM model, ELM-AdaBoost improves the mean accuracy by 5.3 percentage points and shows a smaller standard deviation. A two-tailed paired *t*-test based on the 10-fold cross-validation accuracies further indicates that the improvement of ELM-AdaBoost over ELM is statistically significant (*p* = 0.0027). This result suggests that the AdaBoost ensemble strategy improves the recognition performance of ELM and reduces the influence of random model fluctuations to some extent.

### Ablation Analysis of the Relative Speeding Margin

To further examine whether the relative speeding margin—which is derived from the same P90 statistics that define the speeding label—acts as the sole driver of speeding-category accuracy, an ablation experiment was conducted using the same stratified 10-fold cross-validation protocol. When the relative speeding margin was removed from the input feature set, the overall recognition accuracy of ELM-AdaBoost decreased from 95.8% to 94.1% (a reduction of 1.7 percentage points), and the speeding-category F1-score decreased from 0.98 to 0.95 (a reduction of 2.6 percentage points). The resulting accuracy nevertheless remained well above those of the ELM (90.5%), RF (83.2%), and BP (79.2%) baselines. This moderate rather than catastrophic degradation indicates that the model recovers speeding behavior primarily from the raw kinematic features (vehicle speed, acceleration, and heading dynamics), and that the relative speeding margin provides complementary contextual information rather than functioning as the sole determinant of speeding-category performance. This result mitigates the concern that the classifier merely re-reads the threshold information embedded in its training signal.

To further evaluate the real-time applicability of the proposed method, a computational efficiency analysis was conducted after the classification performance comparison. Since the recognition accuracy of the four models has already been reported in Table 6, this subsection focuses on the training time and single-sample inference time of RF, BP, ELM, and ELM-AdaBoost. The runtime benchmark was performed under the same dataset and experimental environment. Each model was repeatedly tested ten times, and the results are reported as the mean ± standard deviation in Table 7.

As shown in Table 7, the standalone ELM model required the shortest training time because its hidden-layer parameters were randomly generated and only the output weights were analytically solved. The proposed ELM-AdaBoost model required a longer training time than a single ELM classifier because 20 ELM weak classifiers were trained iteratively in the AdaBoost framework. However, its average training time was still less than 1 s. Compared with BP, the proposed model substantially reduced the training time while maintaining higher recognition accuracy, as reported in Table 6.

In terms of inference efficiency, the average single-sample inference time of ELM-AdaBoost was 0.087 ms, which is far shorter than the second-level sampling interval of GPS trajectory data. This indicates that the proposed model can complete behavior recognition before the next GPS record is received. Therefore, although AdaBoost introduces additional iterative computation during training, the proposed ELM-AdaBoost model still satisfies the computational requirements of real-time risky driving behavior recognition.

## 4. Discussion

The proposed ELM-AdaBoost model achieved an average recognition accuracy of 95.8% across five categories of risky driving behavior, outperforming the Random Forest, standalone ELM, and BP neural network models. This demonstrates that combining an ensemble boosting strategy with ELM provides an effective and computationally efficient solution for risky driving behavior recognition. The improvement is particularly evident in speed-related behaviors such as speeding and rapid acceleration, where kinematic features exhibit strong discriminative patterns. In contrast, directional behaviors such as sharp turning and sharp lane changing show relatively lower performance, likely due to overlapping angular velocity characteristics. It should be noted that speeding accounts for a large share of the risky samples (2860 of 5016, approximately 57%; Table 4). This proportion is primarily a product of the relative speeding definition rather than a reflection of disproportionately frequent illegal speeding in actual operation: because the P90 relative threshold flags any vehicle travelling markedly faster than its fleet peers under identical road and time conditions, speed-related exceedances are intrinsically more numerous than the comparatively rare directional events (sharp turning and lane changing). This distribution should therefore be considered when interpreting the per-category performance metrics, as the larger speeding sample provides more training instances for that category.

A key methodological contribution of this study is the use of a P90-based relative thresholding strategy for behavior labeling. Unlike fixed empirical thresholds, the proposed approach derives behavior boundaries from fleet-specific driving distributions, enabling adaptation to real operational conditions. This design is particularly relevant for hazardous chemical transportation vehicles, where regulatory constraints and operational characteristics differ from general traffic conditions.

The validity of the proposed labeling strategy is supported by multiple complementary analyses. First, the derived thresholds are consistent with values reported in prior studies on risky driving behavior. Second, sensitivity analysis across different percentile settings (P85, P90, P95) shows that model performance remains stable, indicating robustness to threshold selection. Third, 120 threshold-identified events were cross-checked using onboard video data, and 89% were judged to be consistent with observed driving maneuvers, providing supplementary observational evidence for label reliability.

The comparison with baseline models further highlights the advantages of the proposed framework. While Random Forest provides stable performance and BP neural networks suffer from class imbalance sensitivity, the ELM-AdaBoost model achieves a better balance between accuracy and generalization. The boosting mechanism also mitigates the instability of randomly initialized ELM models, resulting in improved robustness across all behavior categories.

It should be emphasized that the P90 percentile system functions as a surrogate proxy for driving risk rather than an externally calibrated ground truth. Because the relative speeding margin is derived from the same P90 statistics that define the speeding label, part of the labeling information is structurally embedded in the feature space. The reported accuracy should therefore be interpreted as the model’s ability to reproduce a statistically consistent, fleet-referenced risk criterion, not as validation against externally observed crash outcomes. Similar considerations on the completeness and validation of surrogate-based models in transportation machine learning have been discussed in recent work [29]. In the present study, external validation was limited to the onboard-video cross-check, in which 89% of the inspected events were consistent with observable risky maneuvers; systematic calibration against crash or near-miss records was not possible because no such incident occurred among the labeled events during the monitored period. Establishing the operational validity of the P90 threshold against independent crash or near-miss data is therefore left for future work. To further examine whether the classifier merely re-reads the embedded threshold information, an ablation on the relative speeding margin was additionally conducted (Section Ablation Analysis of the Relative Speeding Margin).

Overall, the proposed framework integrates adaptive label construction and efficient ensemble learning, enabling accurate recognition of risky driving behaviors from GPS trajectory data. The results indicate that the method is suitable for lightweight and real-time driving behavior monitoring in hazardous chemical transportation fleets.

## 5. Conclusions

This study developed a GPS-trajectory-based framework for extracting and recognizing risky driving behaviors of hazardous chemical transport vehicles. By considering speed and direction-related trajectory characteristics, five types of risky driving behaviors were identified, including speeding, rapid acceleration, rapid deceleration, sharp turns, and sudden lane changes. The proposed ELM-AdaBoost model achieved better recognition performance than the comparison models, indicating its potential for lightweight and efficient risky driving behavior recognition.

The practical significance of this study lies in its application to hazardous materials logistics management and transport risk warning. For hazardous chemical transport vehicles, risky driving behaviors may not only increase the probability of traffic accidents but also amplify the consequences of secondary disasters such as leakage, fire, or environmental contamination. Therefore, the proposed recognition method can provide technical support for fleet safety supervision, driver behavior assessment, and real-time risk warning. By identifying high-risk driving events from GPS trajectory data, logistics enterprises and traffic management departments can monitor abnormal driving patterns, issue timely warnings, optimize driver training, and improve the safety management of hazardous materials transportation.

In addition, the proposed method provides a data-driven basis for refined fleet management. Compared with relying only on post-accident investigation or manual supervision, GPS-based risky driving behavior recognition enables continuous monitoring of vehicle operation status and supports proactive safety intervention. The recognition results can be further integrated into transport risk warning platforms to identify high-risk road sections, high-risk time periods, and drivers with frequent risky driving behaviors, thereby improving the prevention and control capability of hazardous chemical transportation risks.

## Figures and Tables

**Table 1:** Detailed information of vehicle trajectory data.

Serial Number	Journey Segment	License Plate Number	Positioning Time	Speed (km/h)	Longitude	Latitude	Direction (°)	Geographic Location
1	1	1	14 October 2023 19:08:31	75	86.128044	46.700121	225	1
2	1	1	14 October 2023 19:08:32	76	86.12785	46.699987	224	1
3	1	1	14 October 2023 19:08:33	75	86.127656	46.699854	225	1
…	…	…	…	…	…	…	…	…
268,621	481	57	30 November 2023 19:15:23	47	83.06709	39.40252	127	366
268,622	481	57	30 November 2023 19:15:30	42	83.0679	39.40208	122	366
268,623	481	57	30 November 2023 19:15:37	24	83.0685	39.40181	124	366

**Table 2:** Sensitivity analysis of percentile thresholds.

Percentile Threshold	Number of Samples in the Constructed Dataset	ELM-AdaBoost Accuracy (%)
P85	9247	94.6
P90	8016	95.8
P95	6825	95.2

**Table 3:** Input features for ELM-based classification.

Feature	Description	Unit
Vehicle speed	Speed of the vehicle at the current GPS record	km/h
Acceleration/deceleration	Speed change between adjacent GPS records	m/s^2^
Heading-angle change	Change in heading direction between adjacent GPS records	°
Angular velocity	Heading-angle change per unit time, calculated according to Equation (3)	°/s
Road-section P90 speed threshold	Relative speeding threshold of the corresponding road section	km/h
Relative speeding margin	Difference between vehicle speed and road-section P90 threshold	km/h

**Table 4:** Class distribution of the final driving-behavior classification dataset.

Class	Number of Samples	Percentage (%)
Normal driving	3000	37.42
Speeding	2860	35.68
Rapid acceleration	521	6.50
Rapid deceleration	610	7.61
Sharp turning	475	5.93
Sharp lane changing	550	6.86
Total	8016	100.00

**Table 5:** Hyperparameter settings of the proposed and baseline models.

Model	Hyperparameter	Value
ELM	Number of hidden neurons	50
	Activation function	Sigmoid
	Input weight initialization	Uniform distribution in [−1, 1]
	Hidden-layer bias initialization	Uniform distribution in [−1, 1]
	Output weight solution	Ridge-regularized Moore–Penrose solution
	Regularization coefficient	$\lambda =1\times 10^{-3}$
ELM-AdaBoost	Number of weak classifiers T	20
	Base classifier	ELM with 50 hidden neurons
	Multi-class strategy	SAMME
	Activation function	Sigmoid
	Input weight initialization	Uniform distribution in [−1, 1]
RF	Number of decision trees	100
	Maximum tree depth	10
	Minimum samples for split	2
	Splitting criterion	Gini index
	Maximum number of features	sqrt
BP	Number of hidden layers	1
	Number of hidden neurons	50
	Hidden-layer activation function	ReLU
	Output-layer activation function	Softmax
	Optimizer	Adam
	Learning rate	0.001
	Maximum number of epochs	1000
	Loss function	Cross-entropy
	Batch size	64

**Table 6:** Classification performance of different models under stratified 10-fold cross-validation.

Model	Category	Accuracy (%)/Mean ± SD	Precision	Recall	F1-Score
RF	Speeding	83.2 ± 1.2	0.98	0.95	0.96
	Rapid acceleration		0.93	0.92	0.92
	Rapid deceleration		0.83	0.81	0.82
	Sharp turn		0.78	0.86	0.82
	Sharp lane change		0.82	0.76	0.79
ELM-AdaBoost	Speeding	95.8 ± 0.7	0.97	0.99	0.98
	Rapid acceleration		1.00	0.97	0.98
	Rapid deceleration		0.96	0.95	0.95
	Sharp turn		0.98	0.90	0.94
	Sharp lane change		0.96	0.95	0.95
ELM	Speeding	90.5 ± 1.0	0.93	0.91	0.92
	Rapid acceleration		0.88	0.89	0.88
	Rapid deceleration		0.92	0.91	0.91
	Sharp turn		0.87	0.83	0.85
	Sharp lane change		0.93	0.90	0.91
BP	Speeding	79.2 ± 1.8	0.93	0.95	0.94
	Rapid acceleration		0.64	0.88	0.74
	Rapid deceleration		0.84	0.85	0.84
	Sharp turn		0.62	0.82	0.71
	Sharp lane change		0.79	0.78	0.78

Note: The reported Accuracy (mean ± SD) is the overall classification accuracy of the model averaged across the ten cross-validation folds, not a per-category accuracy. It is shown once per model and repeated on each behavior row only for table-formatting convenience; the Precision, Recall, and F1-score columns are category-specific.

**Table 7:** Runtime comparison of different recognition models.

Model	Training Time (s)	Inference Time (ms/Sample)
ELM	0.041 ± 0.006	0.006 ± 0.001
ELM-AdaBoost	0.812 ± 0.073	0.087 ± 0.012
RF	1.964 ± 0.142	0.176 ± 0.021
BP	6.483 ± 0.518	0.042 ± 0.006

## Data Availability

The original contributions presented in this study are included in the article. Further inquiries can be directed to the corresponding author.
